# Potential Application Value and Needs of Using Generative AI Videos for Health Management for Older Adults: Qualitative Study

**DOI:** 10.2196/80661

**Published:** 2026-03-09

**Authors:** Ting Liu, Yiming Taclis Luo, Zhenni He, Patrick Pang, Kin Sun Chan, Ying Lau

**Affiliations:** 1 Faculty of Applied Sciences, Macao Polytechnic University, Rua de Luís Gonzaga Gomes, Macao, Macao, 85385993815; 2 Department of Government and Public Administration, Faculty of Social Sciences, University of Macau, Taipa, Macao; 3 The Nethersole School of Nursing, Faculty of Medicine, The Chinese University of Hong Kong, Shatin, New Territories, Hong Kong Special Administrative Region of China, China

**Keywords:** older adults, artificial intelligence, generative AI videos, health management, iterative workshop

## Abstract

**Background:**

Population aging has emerged as a global concern, older adults’ ability to access health knowledge and manage their well-being impacts their health outcomes. In the artificial intelligence era, generative artificial intelligence (GenAI) videos hold promise for enhancing geriatric health management. However, their potential and the needs of older adults in using GenAI videos for health-related purposes deserve a more in-depth investigation.

**Objective:**

This study aims to explore the application potential and multifaceted needs of older adults in using GenAI videos for health information acquisition and management, while providing actionable recommendations for future aging-friendly GenAI video tools.

**Methods:**

A qualitative approach was adopted. Twenty older adults (aged ≥60 y) with basic digital literacy were recruited from communities for participation in iterative GenAI video workshops. Semistructured interviews were conducted. Thematic analysis, following Braun and Clarke’s 6-phase reflexive framework, was used to identify key themes from interview transcripts.

**Results:**

Our results have identified a 3-layer hierarchical structure of needs when older adults interact with GenAI videos. The first layer is technical accessibility, which suggests the direct barriers preventing them from operating GenAI tools independently. The second layer is age-friendly design, which depicts the needs of age-friendly design adaptations, which can help older adults to better use GenAI features. The final layer is integration with professional resources, highlighting the need for integrating professional medical information resources with GenAI tools, so that the GenAI videos can be more appropriately used for health management purposes. By triangulating the core needs of older adults, the capabilities of GenAI, and their remaining gaps, we have found that the existing gaps limit the extent to which the health needs of older adults are met, and also inversely restrain the acceptance and use of GenAI videos by older adults.

**Conclusions:**

This study explored the potential and gaps of using GenAI videos in older adults’ health management. GenAI videos are prominent for self-management of health, but our triangular model reveals coexisting opportunities and challenges. While the core needs drive the development directions of GenAI video technologies, our study suggests that GenAI health videos can benefit from the improvements in technical capabilities, service innovation, localization, and integration of health resources.

## Introduction

The global process of population aging is advancing at an unprecedented rate. United Nations’ data indicate that the global population aged 60 years and older is projected to surge from approximately one billion in 2020 to nearly 2.1 billion by 2050 [1], with the proportion of this age group rising from 13% to 22% [2]. Effective acquisition of health knowledge and adoption of scientific health management strategies impact the quality of life and well-being of older adults [3]. From daily dietary planning and exercise regimen formulation to self-monitoring and management of chronic diseases [4], enhanced health knowledge and management capabilities empower older adults to better understand their health status [5], proactively prevent diseases, and thereby improve their autonomy and social participation [6]. Proper dietary combinations provide adequate nutrition to maintain physiological functions; moderate exercise enhances muscle strength, improves cardiopulmonary function, and optimizes sleep quality [7]. For older adults with chronic conditions (eg, hypertension, diabetes, and cardiovascular diseases), effective health management controls disease progression, reduces complication risks, and lowers medical expenses [8]. Studies indicate that effective health management can decrease the incidence of chronic diseases and associated costs [9]. Older adults actively engaged in health management exhibit better mental health, with lower rates of depression and anxiety [10]. Thus, improving health management among older adults is essential for achieving active aging and intergenerational bonds [11].

In the context of rapid artificial intelligence (AI) advancements, leveraging generative artificial intelligence (GenAI) offers a new technology to assist older adults in health management by enhancing the efficient acquisition of health information and self-management [12]. GenAI, a cutting-edge technology integrating algorithms, such as deep learning and neural networks [13], excels not merely at content generation but, critically, at creating highly personalized, on-demand media. GenAI’s advanced natural language processing [14], data analytics, and learning capacities allow it to transform complex medical knowledge into accessible content [15,16]. Traditional health videos often lack the personalization and real-time adaptability required for complex, individualized health needs [17]. GenAI directly addresses this gap. GenAI-based health videos have emerged as an innovative solution because they can dynamically tailor content based on users’ demographics, specific health conditions, and preferences, for example, generating a short video on “low-sodium recipes for hypertension,” improving relevance and engagement [18]. GenAI videos also enable interactive features, such as personalized recommendations and real-time feedback, which can further support older adults in navigating complex health topics [19]. GenAI videos can generate multilingual and culturally adapted content, reducing barriers for diverse older populations. Despite this immense potential to deliver granular, customized health information, the empirical study of GenAI video adoption and user experience in geriatric health management remains in its infancy.

Despite its potential, older adults face barriers in adopting AI technologies, including the GenAI-based ones. Age-related cognitive decline, manifesting as memory, attention, and reaction time impairments, hinders the understanding and operation of emerging technologies [20]. Neuroscientific research indicates that prefrontal cortex atrophy in older adults reduces multitasking capacity, complicating the execution of complex commands [21]. A historical lack of digital education leaves many unfamiliar with basic operations (eg, searching and installing apps), impeding independent use [22]. Information overload and the proliferation of misinformation further exacerbate challenges in accessing accurate health knowledge [23]. The internet’s rapid, high-volume dissemination overwhelms older users, who struggle to discern reliable content [24]. Misleading health information, often disseminated via sensationalized headlines on social media, exploits these vulnerabilities, leading to misguided decisions [25]. Additionally, technology interfaces often neglect the physiological characteristics (eg, declining vision and hearing) and usage habits of older adults, resulting in poor usability [26]. Most devices and apps feature small fonts, complex icons, and convoluted workflows, rendering them inaccessible to those with sensory impairments [27]. Gesture-dependent interactions and ambiguous feedback mechanisms further increase operational difficulty [28]. Survey data reveal that fewer older adults proficiently use health management apps, with many expressing low trust in GenAI, preferring traditional consultations [29]. This digital divide not only limits GenAI videos’ application in geriatric health management but also underscores urgent needs for age-friendly technological adaptations [30].

Currently, GenAI video applications in geriatric health management remain emergent, with designs and service models inadequately tailored to older adults’ needs, necessitating in-depth research and improvement [31]. Existing tools exhibit unfriendly interaction modes, with content formats and delivery methods failing to accommodate cognitive traits and reading habits [32]. For instance, a study reports that older participants find GenAI videos’ speech interaction too fast and their interfaces overly complex [33]. There is a gap regarding studies that focus specifically on older adults’ interaction experiences, needs, and barriers when using GenAI for health. Against this backdrop, our research aims to investigate older adults’ needs when using GenAI videos for health information acquisition and management, providing targeted recommendations for designing age-friendly GenAI health videos. This research addresses this identified gap in GenAI video applications for geriatric health management, offering evidence-based insights for developers, health care providers, and policy makers to construct human-centric, age-adapted intelligent health systems, ultimately advancing older adults’ health and well-being. Based on these considerations, the following research questions are proposed:

1. What difficulties do older adults face, and what are their needs when using GenAI videos to access health information?
2. What functionalities and services can GenAI videos provide for geriatric health management, and where are the gaps?
3. How can technological design and interaction optimization enhance GenAI video’s applicability and user experience in geriatric health management scenarios?

Addressing these questions will guide future research, offering theoretical and practical foundations for innovative GenAI video applications in geriatric health management. Resolving these issues will facilitate GenAI video’s role in improving health management, narrowing the digital divide, and promoting social equity and harmony.

## Methods

### Participant Recruitment

With the assistance of community partners, we initially recruited 26 older adults residing in nearby communities to participate in an iterative GenAI health video workshop through offline posters. The following inclusion criteria were applied: Participants were aged 60 years or older, possessed basic operational skills with smart devices (eg, smartphones and tablet PCs). Eventually, the results of 20 older adult participants were included in the study. As a qualitative study using thematic analysis, the final sample size of 20 participants was determined based on the principle of thematic saturation. Data collection and analysis proceeded iteratively, and after the 17th interview, minimal new codes or themes emerged, indicating that the core needs and challenges regarding GenAI videos had been sufficiently captured. The subsequent 3 interviews confirmed this saturation point, covering the hierarchical structure of needs. This sample selection was also designed to maximize information power through the deliberate inclusion of participants with diverse demographic factors (eg, varying educational levels and living arrangements) and heterogeneous health needs (eg, hypertension, joint health, and sleep disorders). This deliberate heterogeneity ensured a rich dataset and robust identification of core themes, justifying the sufficiency of the final sample size.

All participants expressed motivation and a need to leverage GenAI and health videos for better self-health management. Participants exhibited diverse health conditions (chronic diseases, healthy, and mild conditions) and living arrangements (eg, living alone, with family, or in care facilities). To clarify the health status definitions: Chronic Disease referred to participants who reported having a long-term medical condition requiring ongoing management, such as hypertension, hyperlipidemia, or diabetes. Mild condition referred to those who reported minor, nonchronic health issues or temporary discomforts that did not impair their daily function. Healthy referred to participants who did not report any diagnosed chronic disease or ongoing medical conditions.

In addition, community workers participated in the research. These professionals are engaged in community health-related work, possess a background in medical knowledge, and have experience assisting older adults with technological tools. Participants voluntarily joined the workshop and did not receive any additional compensation, though tea and snacks were offered. It is important to note that the participant sample, limited to 20 individuals from a single urban context and skewed heavily female (80%), limits the generalizability of the findings and may not fully capture the diverse experiences of the broader older adult population, particularly men and those in rural or low-income settings. We confirmed with the recruiting community workers that this gender imbalance is a common limitation in community-based research, reflecting the higher propensity of female older adults to actively participate in community health and social activities. This limitation is discussed further in the Discussion section.

### Research Design

To reduce older adults’ cognitive load, the study adopted a participatory iterative approach. Each participant received a 15-minute introduction to GenAI to ensure their basic understanding. Before the start of the workshop, a pretest was conducted to collect baseline data, such as participants’ GenAI videos perceptions, technical skills, and self-evaluations of prior knowledge.

The methodology and approach were initially guided by established theoretical models, specifically the Technology Acceptance Model (TAM) [34] and the concept of self-efficacy [35]. We used TAM, which posits that perceived usefulness and perceived ease of use predict technology adoption, to structure the pre and posttest data collection and the iterative refinement process, focusing on understanding factors that influence the acceptance of GenAI-generated health videos among older adults.

**Figure 1.**
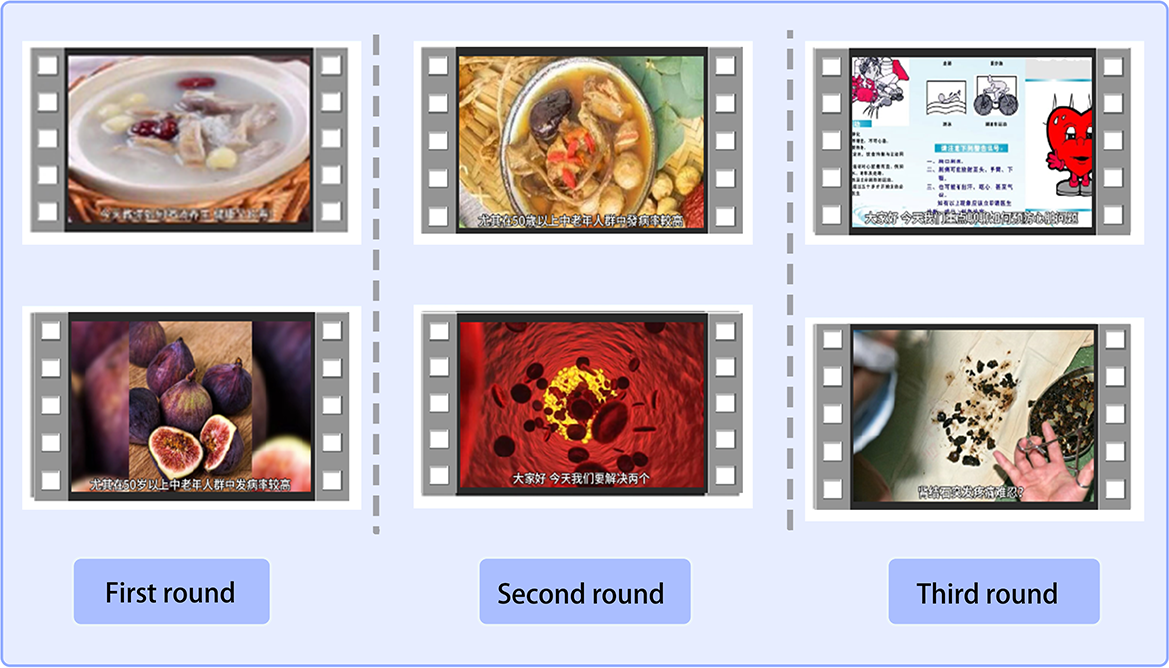
The content and process of generating the videos.

The workshop implementation comprised four steps: (1) Participants proposed a health-related topic that suited their personal health needs. Then, they provided this health topic to GenAI, which converted it into a prompt for generating the GenAI video. They provided the video script generated by GenAI to the video production software CapCut to create the first video. Before generating the video scripts for GenAI, we conducted pretraining on them. We set the generated video scripts to introduce diseases or health issues, solutions and countermeasures, suggested structure generation, and recommended a duration of 3‐5 minutes for short videos to prevent overly long video content from causing cognitive overload for older adults; (2) participants viewed the initial video, discussed their experiences, provided feedback, and proposed new requirements, which were refined into updated prompts for the second video generation; (3) this iterative process was repeated for a total of 3 cycles, culminating in a final optimized video; and (4) after each video generation, community health workers reviewed the content, flagged potential inaccuracies (eg, incorrect medical advice or misleading health claims), and explained the rationale to participants, ensuring discussions were grounded in reliable information. Finally, a posttest was performed to reassess satisfaction, acceptance, and needs after interacting with GenAI videos. The content and process of generating the videos are shown in Figure 1.

### Data Collection and Analysis

Data sources included pre- and posttest interview transcripts. We conducted semistructured interviews in participants’ native language (Cantonese) with audio recording to capture the challenges encountered by the participants and their preferences during the consumption of GenAI videos. The interview transcripts were analyzed with the thematic analysis approach following Braun and Clarke’s 6-phase framework [36]. To ensure data reliability, 2 researchers [TL and PP] independently entered and cross-checked data and resolved discrepancies when disagreements occurred.

### Ethical Considerations

This study was approved by the Human Ethics Committee of Macao Polytechnic University (Approval No.: HEA006-FCA-2025). All participants provided written informed consent before interviews, ensuring comprehension of the study’s purpose, procedures, and potential impacts. All participants have signed an informed consent form before participation. Data were anonymized, with strict confidentiality maintained for personal identifiers. Before workshops, participants were informed that AI-generated content should supplement professional advice, with consent forms emphasizing technical limitations. To reduce the chance of adverse effects caused by misinformation from GenAI videos, community health workers guided the workshops, in which they confirmed the validity of information and stopped the video generation when problems occurred. No compensation was offered to the participants for their involvement in the study.

## Results

### Participant Characteristics

In total, 20 older adults participated (Table 1). Age ranged from 60 to 84 years (mean 71.4, SD 8.16): 8 out of 20 (40%) aged 60‐69, 9 out of 20 (45%) aged 70‐79, and 3 out of 20 (15%) aged ≥80. Gender skewed female (16/20, 80%) versus male (4/20, 20%), possibly reflecting higher female community engagement. Health status: 10 out of 20 (50%) had chronic diseases (hypertension, hyperlipidemia, etc), 8 out of 20 (40%) were healthy, and 2 out of 20 (10%) had mild conditions, highlighting prevalent health management needs. Living arrangements: 9 out of 20 (45%) lived alone, 11 out of 20 (55%) with family, suggesting varying reliance on external support. Education: 7 out of 20 (35%) primary school, 5 out of 20 (25%) junior high, 4 out of 20 (20%) high school, 4 out of 20 (20%) college or university, indicating diverse digital literacy levels. GenAI video experience: 17 out of 20 (85%) were naive users, 3 out of 20 (15%) had limited exposure, ensuring exploration of initial adoption needs. Health topics varied: joint health (n=5), diet (n=4), hypertension (n=3), sleep disorders (n=2), cardiovascular health (n=1), general health (n=2), exercise (n=1), obesity (n=1), and thyroid disease (n=1), reflecting diverse needs and opportunities for GenAI videos’ applications.

**Table 1.** Participant characteristics.

Characteristics		Values (N=20), n (%)
Age (years)		
60‐69		8 (40)
70‐79		7 (35)
≥80		5 (25)
Sex		
Female		16 (80)
Male		4 (20)
Health status		
Chronic disease		12 (60)
Healthy		5 (25)
Mild disease		3 (15)
Living situation		
Living with family		13 (65)
Living alone		7 (35)
Education level		
Primary school or below		9 (45)
Junior high		5 (25)
High school		4 (20)
University or above		2 (10)
GenAI^a^ experience		
No		16 (80)
Yes		4 (20)
Health topics of interest		
Hypertension or hyperlipidemia or hyperglycemia		3 (15)
Sleep disorders		3 (15)
Cardiovascular health		1 (5)
Healthy diet		4 (20)
Obesity		1 (5)
Joint health		4 (20)
Exercise health		1 (5)
Thyroid issues		1 (5)

a GenAI: generative artificial intelligence.

### Thematic Analysis

#### Theme 1: Technical and Accessibility Barriers

Older adults faced foundational challenges in accessing and operating GenAI video tools, stemming from limited digital literacy and interaction difficulties. These barriers highlight a cognitive load required for adoption. Cognitive and digital literacy limitations were prevalent, with participants struggling with fundamental technological tasks. Participant #4 noted, “I have a poor memory and learn new things slowly,” a common reflection of age-related cognitive decline that hinders mastering complex workflows. Participant #12 admitted, “I am illiterate,” revealing the impact of basic educational background on technology adoption and independence. Operational and interface design flaws further compounded these issues, especially given physical challenges. Participant #16 reported, “Poor eyesight affects my typing, so I can only use voice or ask my granddaughter for help,” illustrating a hardware-software mismatch. Participant #14 criticized the “complex button functions” and convoluted workflows that were difficult to follow without prior experience. Consequently, participants often expressed feelings of exclusion, with #13 stating, “GenAI is currently more youth-friendly; it feels designed to make me make mistakes.” Furthermore, localization and support gaps complicated accessibility, such as #4’s explicit request for “Cantonese voice guidance” for better comprehension, and #8’s frustration with having to rely on young family members “for downloading and setting up every new app.” These findings underscore systemic shortcomings in age-friendly interface design and support structures.

#### Theme 2: Trust and Accuracy Expectations

Participants demonstrated a heightened level of caution and demanded rigorous verification regarding the accuracy and reliability of AI-generated health information, indicating a trust deficit. Concerns about misinformation were widespread, with participants often viewing GenAI as a source that requires external validation. Participant #3 expressed direct concerns about being misled, stating, “I worry about being misled by false content from GenAI, especially with medical advice that could harm me.” This led to demands for professional verification and transparency. Participant #14 strongly emphasized the need for human input: “GenAI outputs must be validated by professionals, like a pharmacist or doctor, before I act on it.” Participant #8 reiterated this cautious approach, insisting, “I will consult a doctor before acting on GenAI’s advice; the video is just a starting point.” To build trust, participants requested greater transparency, with #12 advocating for “source citations in AI-generated health information, showing where the medical facts came from,” and #16 suggesting “alerts for abnormal data or warnings if the advice is outside standard guidelines.” This cautious trust stems from their awareness of technological limitations, as #9 cautioned, “GenAI video might misdiagnose my health condition if I input the wrong symptoms.” Despite these concerns, participants acknowledged the medium’s potential, with #7 praising its format: “Videos are more intuitive than text; I can see how an exercise should be done.”

#### Theme 3: Personalized Health Guidance

Participants expressed clear expectations for personalized functionalities that extended beyond generic content, spanning monitoring, risk assessment, and localized solutions. Demands for tailored monitoring and trend analysis were prominent, reflecting needs related to self-management of chronic diseases. Participant #1 requested the tool to provide “automated trend analysis of my blood glucose data over the last 2 weeks, not just today’s number,” while #4 needed specific, daily “calorie, intake calculations for my low-sodium diet.” For risk assessment and nonpharmacological solutions, participants sought customized advice specific to their conditions. Participant #9 inquired about “joint health evaluations based on my walking data,” and #17 sought “nonpharmacological hypertension solutions tailored to my limited mobility.” The need for cultural and localized adaptation was also a key theme, demonstrating that personalization must consider the daily life context. Participant #4 requested “healthy recipe modifications for local Cantonese dishes” to make diet changes practical, and #10 desired “local hospital lookup features integrated into the video advice.” Current GenAI tools fall short of these nuanced expectations. Participant #12 criticized the content as “too-fast, oversimplified video content that doesn’t answer my specific questions,” revealing a gap between generic output and true personalization.

#### Theme 4: Basic Health Management Needs

While GenAI videos demonstrated capability in offering foundational health functions, gaps persisted in terms of depth, integration, and systematic support. Foundational functions and clarity showed progress, such as #3 learning about heart disease basics and #9 understanding osteoporosis risks through visual content. However, limitations were evident in addressing multifaceted health scenarios. Participant #1’s need for combined management of diabetes and cardiovascular concerns went unaddressed by single-topic videos, illustrating a lack of integrated care guidance. Similarly, #9’s need for personalized solutions for concurrent osteoporosis and lung health issues lacked comprehensive guidance due to insufficient data integration. Gaps in professional support were clearly visible, with #14’s request for “thyroid self-check guidance” and #19’s need for a “local hospital locator” being unfulfilled. Consequently, participants often defaulted to traditional support channels, such as #8’s preference for “doctor-confirmed advice” and #15’s reliance on “family assistance*”* to navigate complex information, highlighting the tools’ current inability to serve as a reliable, standalone health resource.

#### Theme 5: Diverse Health Concerns

The older adult population exhibited a highly diversified spectrum of health management needs, necessitating a flexible and broad application scope for GenAI video content. Chronic disease management was a central concern, with #1 explicitly highlighting the need for diabetes prevention, emphasizing blood glucose trend monitoring and personalized dietary recommendations. Health literacy through visualization was sought for complex topics, such as #3, focusing on cardiovascular health and stressing the importance of using GenAI video animations to understand electrocardiogram principles. Needs extended to highly specific daily life and preventative issues, including joint and sleep health, where #15 stated, “I want to understand sleep issues as I have had sleep apnea for over a decade,” and #12 articulated comprehensive requirements for joint pain relief and vitamin supplementation. Furthermore, dietary and safety validation emerged as a distinct concern, with #12 asking, “I heard honeydew melon and eggs are harmful together—is this true?” reflecting a need for rapid validation of common health myths. This diversity confirms that GenAI videos must shift from single-disease treatment to comprehensive, highly specialized prevention and daily health information services in geriatric health management.

The abovementioned themes were summarized into three high layers of needs, representing the needs of (1) technical accessibility, (2) age-friendly design adaptations, and (3) integration with professional medical information resources. Table 2 lists the layers, themes, and subthemes described in this subsection.

**Table 2.** Summary of results of thematic analysis.

Layers, themes, and subthemes		Key insights from interview texts
Layer 1: Technical accessibility		
	Technical and accessibility barriers: digital literacy limitations and language and localization gaps	Inability to operate tools independently (eg, typing, navigating interfaces) Insufficient Cantonese support and local specific health resources
Layer 2: Age-friendly design adaptations		
	AI usage barriers: digital literacy challenges and interface simplicity needs	Difficulty in operating complex interfaces due to poor memory or illiteracy Demand for voice interaction, large fonts, and step-by-step guidance
	Trust and accuracy expectations: cautious trust in AI^a^ and localization and age-friendly features	Openness to try but requiring human verification for critical decisions Preference for Cantonese voice, localized services, and age-adapted design
Layer 3: Integration with professional medical information resources		
	Personalized health guidance: dynamic health monitoring, visualized knowledge delivery, and multimodal interaction	Autogenerated reports (eg, blood glucose trends and sleep reports) Animated videos explaining medical concepts (eg, ECG^b^ and osteoporosis) Voice-guided operations and large-font interfaces
	Basic health management needs: symptom identification, nonpharmacological solutions, professional medical diagnosis, and critical decision-making	Early warning for osteoporosis, cardiovascular risks, and digestive issues Sleep improvement tips and joint protection exercises Inability to replace doctors for definitive diagnoses (eg, osteoporosis and sleep apnea) Lack of human verification for health suggestions (eg, medication and surgery)
	Diverse health concerns: chronic disease management, joint health and injury prevention, sleep health improvement, and digestive health and dietary safety	Need for personalized monitoring and guidance on hypertension, diabetes, and cardiovascular diseases Guidance on joint protection exercises and fall prevention during travel Nonpharmacological solutions for insomnia and sleep apnea Personalized dietary recommendations and food compatibility validation

a AI: artificial intelligence.

b ECG: electrocardiogram.

## Discussion

### Principal Findings

**Figure 2.**
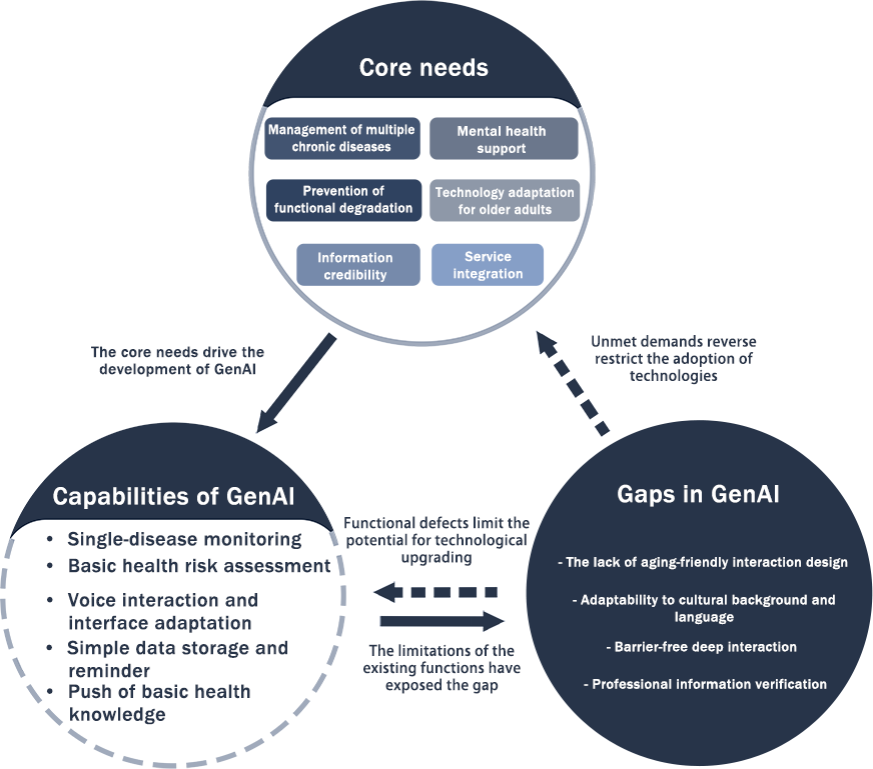
Dynamic relation of the needs, capabilities, and gaps of generative artificial intelligence videos. GenAI: generative artificial intelligence.

The results demonstrate the interactive relationship among the needs of older adults in using GenAI videos for health management, the capabilities of GenAI videos, and the existing gaps in GenAI video tools, as synthesized in Figure 2. The core needs of older adults drive the development and application direction of GenAI video technology, promoting the realization of its functions in areas such as health monitoring and information interaction. However, the current capabilities of GenAI video tools reveal unmet gaps in areas such as multidisease integration and professional medical diagnosis. On one hand, these gaps directly limit the extent to which the health needs of older adults are met; on the other hand, they also inversely restrain the further acceptance and use of GenAI video technology by older adults. As such, this forms a dynamic triangular relationship that requires more attention among user needs, GenAI capabilities, and the gaps to be filled.

This closed loop shows that the digital transformation in the field of geriatric health management is not a linear process of progress but rather a complex relationship where the potential of technological empowerment coexists with the challenges of age-friendly adaptation. Achieving sustainable alignment between technological development and user needs requires systematic design to balance efficiency and humanistic care. Table 3 also provides a detailed list of the specific context regarding the needs of older adults, the capabilities of GenAI video, and the gaps in GenAI video, which highlights the multidimensional characteristics and the mismatch of needs of current tools.

**Table 3.** The comparison of the needs, capabilities, and gaps of generative artificial intelligence videos.

Core dimension	Core needs	Capabilities of GenAI^a^	Gaps in GenAI
Health needs coverage	Comprehensive management of multiple chronic diseases, personalized health guidance, and mental health support	Single-disease monitoring (eg, blood glucose) and basic health risk assessment	Integrated management of multiple diseases, professional medical diagnosis, and solutions for complex health issues
Technical adaptability	Simple operations, cultural adaptation (dialect or localized content), and barrier-free interaction	Basic voice interaction and large-font interface	Fully text-free operation, automated complex tasks, and deep cultural customization
Information interaction	Authoritative and trustworthy information, personalized recommendations, and human verification channels	Basic information prompts and simple data recording	Medical-grade information verification, dynamic credibility rating, and real-time human validation
Service integration	Integration of medical, family, and community resources and end-to-end health management	Health data storage and basic consultation services	Real-time integration with medical systems and family-community resource network

a GenAI: generative artificial intelligence.

The health needs of older adults show a trend toward diversification and refinement, covering multiple areas, such as chronic disease management, prevention of functional decline, and mental health support, with highly heterogeneous combinations of needs among different individuals. At the level of technology use, insufficient digital literacy and interface interaction barriers constitute the main barriers to use, manifesting as fear of complex operations, dependence on clear guidance, and an urgent demand for culturally adapted features. In terms of trust, older adults exhibit a conservative attitude, recognizing the efficiency advantages of GenAI in information presentation and basic services while maintaining reservations about the reliability of professional medical advice and emphasizing the importance of human verification and professional support. Regarding functional supply, existing GenAI video tools have made initial progress in standardized service areas, such as basic health monitoring and visualized knowledge delivery, but they still show deficiencies in the depth of personalized adaptation, the ability to handle complex scenarios, and the level of integration with professional medical services.

### Multidimensional Needs of Older Adults Using GenAI Video for Health Management

The core needs of older adults for GenAI-driven health management exhibit a hierarchical structure, which can be systematically deconstructed into 3 progressive dimensions. The first layer focuses on technological accessibility and operational inclusivity, manifesting as urgent demands for simplified interaction workflows [37], enhanced multimodal interactions [38], and interface designs adapted to physiological decline [39]. These needs stem from the structural deficiencies in digital literacy among older adults, characterized by barriers in comprehending complex technical logic and heightened sensitivity to operational memory load [40]. This cognitive load is not merely an individual failing but a manifestation of the digital divide, wherein technologies often neglect the specific cognitive and physical requirements of older users, reflecting ageism in design standards.

The second layer emphasizes personalized and precise health management adaptations, encompassing dynamic risk assessments based on longitudinal health data, culturally sensitive content delivery, and cross-scenario health guidance. This layer reflects a paradigm shift from passive acceptance to active participation in health management.

The last layer centers on professional medical support and system integration capabilities, including disease risk prediction models approaching clinical decision-support levels, interoperable health data exchange across medical institutions, and integrated emergency response systems. Such needs highlight a functional expectation shift from “information assistance” to “decision participation” for GenAI video tools. The corresponding challenges arise from multifaceted technical and social constraints. On the technical front, digital divides manifest as high learning costs, lack of age-friendly design, and complex functional logic [41]. Socially, these challenges are reflected in the lagging digital transformation of medical trust systems [42], fractured intergenerational digital support networks [43], and uneven regional health resource distribution [44]. The interplay between these needs and barriers underscores a structural mismatch in digital transformation. Specifically, the contradiction between standardized technological supply and personalized health demands, as well as the imbalance between digital access opportunities and capacity building. The persistent reliance on human verification is closely tied to low eHealth literacy, where users lack the critical skills to evaluate information quality, amplifying a lack of trust in nontraditional sources such as GenAI.

### Capabilities of GenAI Video for Older Adults’ Health Management

GenAI video has achieved foundational breakthroughs in older adults’ health management across 3 interconnected dimensions. First, information delivery efficiency has improved [45], with natural language processing and multimodal interactions transforming medical knowledge into accessible formats, such as animated explanations and voice narrations, thereby lowering health information acquisition barriers [45]. Second, the level of service automation has increased, as big data analytics and rule-based engines enable health monitoring, basic risk assessments, and standardized recommendations [46]. Third, accessibility has been enhanced through cloud computing and mobile internet technologies, transcending temporal and spatial constraints [47]. These advancements have facilitated a shift from experience-driven to data-driven health management. However, the operational scope of GenAI video tools remains generic, while video generation with complex information or expert knowledge still requires human guidance [48]. GenAI videos’ role remains tool-oriented, and people tend to use them for enhancing efficiency rather than replacing professional medical opinions.

### Gaps of GenAI Video for Older Adults’ Health Management

Current applications of GenAI videos exhibit limitations that require breakthroughs in several areas. From the health care perspective, there is not enough integration of accurate health information in managing complex chronic diseases [49]. Regarding emotional interaction, the system shows a weak capacity to interpret unstructured health demands, such as ambiguous expressions, or provide empathetic responses [50]. Concerning system integration, barriers to interoperability within health care ecosystems persist [31]. Additionally, the cultural sensitivity of GenAI video tools remains insufficient, with inadequate adaptation to regional variations and traditional health beliefs, such as limited support of different spoken languages [51]. Technologically, it is essential to develop lightweight, age-friendly solutions, such as offline voice assistants and multimodal sensors, and enhance algorithm transparency. Regarding service models, exploring “AI+human expert” hybrid collaboration mechanisms will be crucial. Policy-wise, establishing cross-sector digital health standards, including data security protocols and service quality metrics, is necessary. Socially, activating family-community-health care collaboration networks will foster an inclusive ecosystem. The improvement strategies involve constructing a collaborative framework across technology, health care, and social domains. On the health care side, establishing clinical validation standards for AI-assisted diagnostics is crucial [52]. From the societal point of view, developing intergenerational digital support networks and community-based digital literacy programs will be necessary [53]. These gaps, particularly the lack of integrated, verified professional support, reinforce the existing digital divide by making the tool unreliable for those with lower health literacy, potentially deepening health inequities. These measures will facilitate the evolution of GenAI videos from a “basic service” to a “comprehensive health management partner.”

### Practical Implications

Based on the identified hierarchical needs and capability gaps, our findings offer concrete, actionable recommendations for key stakeholders to enhance the real-world applicability of GenAI health videos. For AI developers and designers, this means implementing mandatory Age-friendly interface design principles, prioritizing simplified, one-step workflows to reduce cognitive load, large-font visual interfaces, and enhanced multimodal interactions, especially high-accuracy dialect-supported voice control to overcome digital literacy barriers. Developers must also integrate transparent features showing the data source and reliability score for all medical claims. In parallel, health care organizations and policy makers should establish clinical vetting standards and certification processes for AI-generated health content to bolster trust. Policy makers should mandate the development of interoperable health systems that allow secure, real-time data exchange between GenAI tools and eHealth records, enabling true personalized, integrated chronic disease management. Community and social support networks have a crucial role in investing in community-based digital literacy programs that specifically teach older adults how to critically evaluate AI-generated health information, rather than just teaching basic operations. Implementation guidelines should focus on hybrid models where community health workers act as the “human verification” bridge between the AI tool and the end user.

### Ethical Considerations in GenAI Video for Older Adults’ Health Management

The application of GenAI videos in geriatric health management raises complex ethical issues that necessitate a comprehensive framework across technological design, service delivery, and policy regulation [54]. First, the balance between informed consent and autonomy presents a core challenge. Cognitive decline may hinder older adults’ understanding of AI risks, while the over-reliance on proxies risks infringing their autonomy [55]. Solutions include developing visualized consent processes and clearly defining human-AI responsibility boundaries [56]. Second, algorithmic fairness and health inequities require urgent attention. Training data biases may exacerbate disparities, while hardware limitations could exclude economically disadvantaged groups [57]. Mitigation strategies involve privacy-preserving data integration and promoting affordable, age-friendly devices [58]. Third, the conflict between data privacy and generational security is prominent [59]. Finally, ambiguity in responsibility attribution challenges traditional medical ethics. Errors in AI-generated advice involve multiple stakeholders, including developers, clinicians, and users, necessitating decision-tracking systems and legislation for scenario-specific accountability [60]. These challenges underscore the need for a governance framework that balances efficiency, equity, and security through interdisciplinary collaboration.

### Recommendations for Future Research

The sustainable development of GenAI videos for older adults’ health management requires integrated strategies across multiple dimensions, which sheds light on the directions of future research. The ultimate goal is to create an inclusive health ecosystem where GenAI video empowers rather than replaces human care. Future research should focus on designing adaptive interfaces that accommodate the diverse digital literacy levels of older adults. This includes developing progressive disclosure mechanisms and context-aware guidance systems to reduce cognitive load, alongside iterative usability testing with age-diverse cohorts to refine interaction paradigms. From a gerontological standpoint, longitudinal studies are needed to map the dynamic evolution of older adults’ digital competencies, identifying age-related changes in technology adoption patterns, generational differences, culturally tailored content, and community-based digital literacy programs. For computer science researchers, the priority lies in advancing GenAI algorithms to enhance personalization accuracy. By integrating insights from these 3 domains, we can develop GenAI video systems that not only optimize technological efficiency but also embed humanistic values, ultimately fostering active aging through inclusive, empowering, and sustainable digital solutions.

### Limitations

We acknowledge several limitations in this work. First, the single-point-in-time design limits our ability to capture the dynamic changes in older adults’ digital literacy and GenAI acceptance over time; the barriers identified here may change rapidly as users gain experience, and a longitudinal study would be necessary to map the learning curve and long-term acceptance trajectory. Second, the sample primarily comprised urban, middle- to high-income older adults, with a notable skew toward female participants. While this skew reflects higher community engagement among this demographic, it may overestimate the average digital literacy level and underrepresent the unique health management needs and severe technological barriers faced by older adults in rural or low-income settings, particularly men. Finally, the qualitative nature of this study provides rich, deep insights into user needs but limits the statistical generalizability of the findings. While these limitations persist, they provide valuable directions for future research.

### Conclusions

Based on a series of iterative workshops, this study systematically explored the opportunities and challenges of using GenAI videos for older adults’ health management, yielding the following key conclusions. First, the needs of older adults for GenAI video exhibit a clear hierarchical structure: their fundamental needs focus on technological accessibility, then move to the emphasis of personalized adaptation, and the highest layer of needs prioritizes the integration with professional health information. The study confirms that GenAI videos have demonstrated potential in geriatric health management, notably enhancing information delivery efficiency, lowering the barriers of health knowledge acquisition, and transcending temporal and spatial constraints of health management. However, notable gaps remain between the features of GenAI video tools and older adults’ needs, particularly in the depth of personalization, the capabilities of complicated responses, localization, as well as the accuracy and reliability of medical knowledge. The value of our research lies in recognizing the advantages and limitations of GenAI videos in this context, which can shed light on future research and solutions that can benefit older adults.

## Abbreviations

AI: artificial intelligence
GenAI: generative artificial intelligence
TAM: Technology Acceptance Model
